# Character

**Published:** 1871-12

**Authors:** 


### HALL'S

### JOURNAL OF HEALTH.

Vol. XVIII. — DECEMBER, 1871. — No. XII.

### CHARACTER.

When the Harper Brothers were burned out a few years ago, losing about a million of dollars, even while their immense building was but half consumed, with its incalculable treasures of books, business men, who knew their sterling integrity, came to them and offered them all the money they wanted to enable them to begin again. Perhaps this was the fruitage of the seed sown by their noble-hearted mother, when they were boys. It fell on this wise. There was an old-fashioned schoolmaster in the neighborhood, whose custom it was to board around among the parents of his pupils, in part payment of tuition bills; in the course of years he fell into drinking habits, which increased upon him, until at length he became incapacitated for the performance of his official duties and was deposed, when he soon became so offensive in manners and dress and conversation, that he was excluded even from the kitchens of the neighborhood.

One dreary winter's day Mrs. Harper's eye fell upon him, and her heart was so much moved at his forlorn and outcast condition that she determined on the instant to try and save him, brought him to her house, allowed him to warm himself by the kitchen fire, gave him a good dinner, replaced his filthy rags with clean and comfortable clothing; put him in the way of doing something for himself; stood by him; encouraged, countenanced him, always meeting him with a smile of recognition. By degrees he began to feel that he was a man again; he felt proud of Mrs. Harper's speaking to him, and determined to be worthy of her good opinion; went to meeting, eventually joined the Methodist Church, and soon thereafter died in the triumph of the faith which its simple doctrines inspire. It makes one's heart bleed to think how many mother Harpers are needed nowadays to help the fallen, men and women both; to take them by the hand, and by kind words, and tender deeds, and judicious help, to show them a better way; millions of immortals could be saved for earth and heaven too; but where are the mother Harpers to be the instruments of such salvation? They would be paid good wages, not only in their own persons, but good deeds bring blessings by inheritance, descending even to children's children.

But a poor man's character is more valuable to him than to a millionaire; because if the rich man loses his good name he has his money to back him, and he lives on; but the poor man must perish.

Within a few months a man of seventy-five years, living near New York city, was accused of having murdered another who had worked for him twenty-one years before, who had suddenly disappeared, and had never been heard of since. He was arrested. He was a poor man, and old, and had few friends, none who were able to help him to money to procure counsel to attend his case; to be accused of murder in his old age, perhaps to hang for it, because he had not the means to search out evidence to clear him — it was more than human nature could endure, and he fell down dead. In less than a month later, the man who was supposed to have been murdered was found to be alive and well. Slander kills.

More than fifty years ago a young man lived in a Western city, and, as a druggist, was accumulating property, possessing the respect and confidence of the community, as was proved by the fact that, as he was about starting to the East to lay in stock, the cashier of a bank handed him a package of money in bills to be handed to a bank officer in Philadelphia; being very obliging, he received the package, and promised to deliver it promptly on his arrival, which he did; the cashier of the bank to whom he delivered the bills looked over them hastily, placed them in a drawer, saying it was "correct," and went on with his writing.

A month later the Western banker came to the young druggist and informed him that a bill of a large denomination was missing. The young man said he did not know how that could be, for he had delivered the package as he had received it, that the Eastern banker had looked it over, pronounced it correct, and that he thought his responsibility ended there. The facts stood thus: two prominent business men, in responsible positions, on one side, and the unsupported say so of a young druggist on the other; the odds were too unequal, and the young man had to go to the wall; the community withdrew their patronage and their confidence; his business was broken up; he first attempted one thing, then another, but a cloud seemed to hang over him. Years rolled on. The story was handed down from one to another, and new comers imbibed the prejudices of the old; and twenty years later there was an odium attached to his character, so that at the mention of his name there was that falling of the countenance which meant, "no confidence." The young druggist became an old man, but never succeeded in regaining the social position he had lost. He died in Coventry. The old desk was taken to a shop to be repaired. On removing the drawer, the missing bank bill was found to have been lodged behind it! In the light of facts like these, it is clear that in a business point of view —

### CHARACTER IS EVERYTHING.

It is a man's capital, his living, his meat and bread; and yet there seems to be in almost all persons a kind of pleasure in hearing and retailing what is prejudicial to others, our neighbors, our associates, and even our friends; it is only when the venomous tongue touches our kith and kin, and thereby touches us, that we recoil at the slightest puncture. We cannot well avoid listening to the defamation of others, although there are some, a few

### STERLING SOULS

who, on the instant of hearing the first venomous word against the absent, find or make occasion to quit the company. But it ought to be easy for any one of magnanimity, of just and generous impulses and honorable feelings, to avoid lending himself to the commission of a social crime, worse than highway robbery, worse than midnight theft,—the crime of spreading a defamation by its repetition, whether by word or sign, direct or indirect, unless called upon to do so in a court of justice, or to guard one's friends or kindred from pecuniary injury; under any other circumstances, no man or woman has any right to utter, or write, or even hint, what would cast a stain upon the character of any absent person, even if personally known to be true. But to do such a thing on the representation of third persons, however high the character of that third person might be, is meaner than theft; because a thief may feel himself driven to it by his necessities, and gains something by it — may help to save himself, or wife and children, from starving; but the social thief steals to the irreparable injury of friend and stranger alike, without any possible benefit to himself, beyond the despicable gratification of a tattler and talebearer.

The coward on the battle-field is instinctively execrated by the common consent of mankind, savage as well as civilized; but the moral coward, who says of the absent what he would be ashamed and afraid to repeat if present, is the most pitiable of his kind. This article will not have been written in vain if a single reader resolves, that for himself he at least will not "fall into the same condemnation," especially as it is written, "For without are . . . . whoremongers, and murderers, . . . . and whosoever loveth and maketh a lie."

